# Association of the I264T Variant in the Sulfide Quinone Reductase-Like *(SQRDL)* Gene with Osteoporosis in Korean Postmenopausal Women

**DOI:** 10.1371/journal.pone.0135285

**Published:** 2015-08-10

**Authors:** Hyun-Seok Jin, Jeonghyun Kim, Sangwook Park, Eunkuk Park, Bo-Young Kim, Vit-Na Choi, Young-Hyun Yoo, Bom-Taeck Kim, Seon-Yong Jeong

**Affiliations:** 1 Department of Biomedical Laboratory Science, College of Life and Health Sciences, Hoseo University, Asan, Republic of Korea; 2 Department of Medical Genetics, Ajou University School of Medicine, Suwon, Republic of Korea; 3 Department of Biomedical Sciences, Ajou University Graduate School of Medicine, Suwon, Republic of Korea; 4 Department of Biomedical Laboratory Science, College of Health, Kyungwoon University, Gumi, Republic of Korea; 5 Division of Intractable Disease, Center for Biomedical Sciences, National Institute of Health, Korea Centers for Disease Control & Prevention, Cheongju, Republic of Korea; 6 Department of Anatomy and Cell Biology and Mitochondria Hub Regulation Center, College of Medicine, Dong-A University, Busan, Republic of Korea; 7 Department of Family Practice and Community Health, Ajou University School of Medicine, Suwon, Republic of Korea; Children's National Medical Center, Washington, UNITED STATES

## Abstract

To identify novel susceptibility variants for osteoporosis in Korean postmenopausal women, we performed a genome-wide association analysis of 1180 nonsynonymous single nucleotide polymorphisms (nsSNPs) in 405 individuals with osteoporosis and 722 normal controls of the Korean Association Resource cohort. A logistic regression analysis revealed 72 nsSNPs that showed a significant association with osteoporosis (*p*<0.05). The top 10 nsSNPs showing the lowest *p*-values (*p* = 5.2×10^-4^–8.5×10^-3^) were further studied to investigate their effects at the protein level. Based on the results of an *in silico* prediction of the protein’s functional effect based on amino acid alterations and a sequence conservation evaluation of the amino acid residues at the positions of the nsSNPs among orthologues, we selected one nsSNP in the *SQRDL* gene (rs1044032, SQRDL I264T) as a meaningful genetic variant associated with postmenopausal osteoporosis. To assess whether the SQRDL I264T variant played a functional role in the pathogenesis of osteoporosis, we examined the *in vitro* effect of the nsSNP on bone remodeling. Overexpression of the SQRDL I264T variant in the preosteoblast MC3T3-E1 cells significantly increased alkaline phosphatase activity, mineralization, and the mRNA expression of osteoblastogenesis markers, *Runx2*, *Sp7*, and *Bglap* genes, whereas the SQRDL wild type had no effect or a negative effect on osteoblast differentiation. Overexpression of the SQRDL I264T variant did not affect osteoclast differentiation of the primary-cultured monocytes. The known effects of hydrogen sulfide (H_2_S) on bone remodeling may explain the findings of the current study, which demonstrated the functional role of the H_2_S-catalyzing enzyme SQRDL I264T variant in osteoblast differentiation. In conclusion, the results of the statistical and experimental analyses indicate that the SQRDL I264T nsSNP may be a significant susceptibility variant for osteoporosis in Korean postmenopausal women that is involved in osteoblast differentiation.

## Introduction

Osteoporosis is a common skeletal disease characterized by a decreased bone mass, resulting in an enhanced risk of bone fracture [1, 2]. Osteoporotic fractures are a major concern in health care because of associated morbidity and mortality, particularly in elderly women and men [2, 3]. Besides age-related bone loss affecting both women and men, estrogen deficiency caused by the menopause is critical to the pathogenesis of osteoporosis in women [1, 4]. Multiple genetic and environmental factors influence the pathogenesis of osteoporosis [5, 6]. Despite the high heritability of the maximal amount of bone mineral density (BMD) gained during growth and other determinants of fracture risk, including the properties of bone, as demonstrated in ultrasound twin and family studies [7, 8], the genetic factors regulating BMD levels and the susceptibility to osteoporosis in the general population are largely unknown [7, 9, 10].

In the past two decades, robust candidate-gene approach association studies, genome-wide association (GWA) studies, and meta-analyses have been conducted to identify the genetic variations that are associated with bone mass, BMD, osteoporosis, and/or osteoporotic fracture. These studies have uncovered numerous putative single-nucleotide polymorphisms (SNPs) in many genes/loci [7, 11–14]. At this time, there are 988 genes listed in the HuGe Navigator database (http://hugenavigator.net) associated with osteoporosis. Among these, 122 genes have been reported in GWA studies. In addition, 148 genes are listed as postmenopausal osteoporosis-associated genes. The vitamin D receptor genes (*VDR*), estrogen receptor 1 (*ESR1*), and collagen type 1 alpha 1 (*COL1A1*) genes are the top-three reported genes in research studies of many ethnic cohorts in the HuGe Navigator database. Considering the high heritability (*h* ^2^, 25–85%) of osteoporosis-related phenotypes, including BMD and fracture [8], the vast majority of genes/loci of susceptibility for osteoporosis and/or osteoporosis-related traits remain to be identified.

GWA studies are very effective for discovering novel genetic variants influencing disease and traits, but they also have considerable limitations, including the potential for false-positive and false-negative results caused by biases related to the selection of study participants and SNP genotyping errors [15]. It is difficult to validate the causal biological roles of GWA findings if the discovered SNPs are located on regions of noncoding introns and no known genes [15]. Data from GWA studies for human diseases and traits revealed that only approximately 12% of disease and/or trait-associated SNPs are located in protein-coding regions of genes, whereas approximately 40% of disease and/or trait-associated SNPs are located in noncoding intron regions of genes and intergenic regions [16]. Similarly, most SNPs associated with osteoporosis and/or osteoporosis-related traits in the HuGe Navigator database and the literature are located in noncoding intron regions of genes [17].

Nonsynonymous SNPs (nsSNPs) that are located within the coding region of genes cause alterations in the amino acid sequence of proteins. These nsSNPs can directly or indirectly affect the corresponding protein functions and interactions due to protein misfolding, improper protein modification, such as phosphorylation and sumorylation, and other functional consequences of protein modifications [18]. As nsSNPs are believed to exert potential functional effects on cellular function and gene-gene networks, ultimately resulting in disease, an association study of nsSNPs may identify genuine susceptibility variants that satisfy not only the requirement of statistical significance but also of association reliability.

In this study, we aimed to find novel nsSNP(s) associated with postmenopausal osteoporosis by a systematic scanning approach. We performed a GWA study of 1180 selected nsSNPs for osteoporosis in 1,127 Korean postmenopausal women. Based on the results of a statistical analysis and *in silico* prediction analyses, one nsSNP in the *SQRDL* gene was selected as a primary candidate for a further replication study. We demonstrated experimentally the functional role of the discovered *SQRDL* nsSNP in osteoblast differentiation.

## Materials and Methods

### Study subjects in the association study

The study cohort, the Korean Association REsource (KARE) cohort, has been described in a previous report [19]. Briefly, 8,842 participants (4,183 men and 4,659 women) were recruited from two community-based epidemiological cohorts in Ansung and Ansan cities as part of the Korea Genome and Epidemiology Study (KoGES). Among the 4,659 female participants, 1,412 postmenopausal women (age 50–69) who had not received any pharmaceutical medications were selected. The study subjects in this study included osteoporosis cases (*n* = 405) and controls (*n* = 722), and the remaining 285 women with osteopenia were excluded. The basic characteristics of the study subjects, including their body mass index (BMI) and bone properties, are described in Table 1. The mean ages, mean BMI, mean values of the distal radius speed of sound (DR-SOS), mean values of the DR-SOS T-score, mean values of the midshaft tibia speed of sound (MT-SOS), and mean values of the MT-SOS T-score were significantly different between the osteoporosis cases and normal controls.

**Table 1:** Basic characteristics of the postmenopausal women in the association study.

Characteristics	Cases	Controls	*p*-value*
No.	405	722	
Age (year)	61.84 ± 4.70	56.98 ± 5.47	< 0.0001
Body mass index (BMI) (kg/m2)	25.18 ± 3.28	24.54 ± 2.93	0.0013
Distal radius speed of sound (DR-SOS) (m/s)	3972 ± 185	4224 ± 146	< 0.0001
Midshaft tibia speed of sound (MT-SOS) (m/s)	3600 ± 115	3920 ± 105	< 0.0001
DR-SOS T-score	−1.72 ± 1.57	0.41 ± 1.21	< 0.0001
MT-SOS T-score	−3.33 ± 1.11	−0.26 ± 0.96	< 0.0001

*Significant differences in the characteristics between the controls and cases were determined by the two-tailed Student’s *t*-test. Abbreviation: *p*-value, probability value.

The bone speed of sound (SOS) value was measured at the DR and midshaft tibia using the Omnisense 7000P quantitative ultrasound (QUS) device (Sunlight Medical Ltd, Tel-Aviv, Israel) to assess bone status [20]. Bone density and elasticity influence the bone SOS value. The T-score was calculated by dividing the difference between the measured SOS and the mean SOS in a healthy adult population by the standard deviation (SD) of SOS in an adult population. Subjects in whom T-scores at either the DR-SOS or MT-SOS were <−2.6 SD and −3.0 SD, respectively, were considered case subjects to distinguish between case and control subjects, according to the diagnostic categories established for adult women [21]. Subjects in whom both the DR-SOS and MT-SOS T-scores were >−1.4 SD and −1.6 SD, respectively, were considered control subjects [21, 22]. This study was approved by the institutional review board of the Korean National Institute of Health. Written informed consent was obtained from all subjects.

### SNP genotyping, quality control, and nsSNP selection

The genotype data were provided by the Center for Genome Science, the Korea National Institute of Health. The detailed genotyping and quality control processes have been described in a previous report [19]. Briefly, most DNA samples were isolated from the peripheral blood of participants and genotyped using the Affymetix Genome-Wide Human SNP array 5.0 (Affymetrix; Santa Clara, CA, USA). The accuracy of the genotyping was calculated by Bayesian Robust Linear Modeling using the Mahalanobis Distance (BRLMM) genotyping algorithm [23]. Samples that had lower genotyping accuracies (≤ 98%), high missing genotype call rates (≥4%), high heterozygosity (>30%), or gender biases were excluded from this study.

Using the web-based BioMart data mining tool (version 5.0) (http://www.ensembl.org/biomart/), we selected 1,180 nsSNPs from the quality control-passed 352,228 SNPs, on the basis of the information in the SNP database (dbSNP) version 129, as described in a previous report [24].

### *In silico* prediction of functional effects of nsSNPs

*In silico* prediction of the functional effects of the nsSNPs at the protein level was analyzed using the Sorting Intolerant From Tolerant (SIFT) program and the Polymorphism phenotyping v2 (PolyPhen-2) program [18, 25]. The SIFT program predicts “damaging” or “tolerated” functional effects of amino acid changes, and the PolyPhen program predicts three categories according to deleterious effect levels: probably damaging, possibly damaging, or benign.

### Construction of *SQRDL* cDNA clones

A human *SQRDL* cDNA clone was provided by the Korea Human Gene Bank, Medical Genomics Research center, KRIBB, Korea (http://genbank.kribb.re.kr). An amino acid changed *SQRDL* cDNA clone at the 264 residue (SQRDL_I264T) was constructed using a Quikchange Lightning site-Directed Mutagenesis Kit (Agilent Technologies; California, CA, USA) according to the manufacturer’s instructions. The human SQRDL wild-type cDNA and human SQRDL I264T cDNA were inserted into a pCDH-CMV-MCS-EF1-Puro expression lentiviral vector (System Biosciences; California, CA, USA). The constructs were transfected into 293TN cells and incubated for 72 h, and then the supernatants containing viral particles were collected by centrifugation and stored at 4° C for further experiments.

### Cell culture

All cells used were grown in α-MEM medium (Life Technology; Carlsbad, CA, USA), supplemented with 10% fetal bovine serum (Sigma-Aldrich; St. Louis, MO, USA), 100 U/mL of penicillin (Duchefa; RV Haarlem, Netherlands), and 100 μg/mL of streptomycin (Duchefa), and incubated in a humidified atmosphere at 37° C and at 5% CO_2_.

Mouse preosteoblast MC3T3-E1 cells were purchased from the RIKEN Cell Bank (Tsukuba, Japan). The MC3T3-E1 cells with passages 5–10 (after purchase) were used for all experiments. For osteoblast differentiation, the MC3T3-E1 cells were cultured in the same medium, supplemented with 50 μg/mL of ascorbic acid and 10 mM of β-glycerophosphate for 7–14 days, and the medium was changed every 3 days.

To prepare primary-cultured monocytes, the bone marrow of femoral bones of 6-week-old mice was removed by flushing with a fine-bore syringe into α-MEM medium in the presence of 1 mM of ascorbate-2-phosphate (Sigma-Aldrich). Primary-cultured monocytes were validated by immunophenotypic analysis with a CD11b antibody (BioLegend; San Diego, CA, USA) using the FACS Aria III cell sorter (BD Biosciences; San Jose, CA, USA) and FACS Diva software (BD Biosciences). For osteoclastogenesis, primary monocytes were cultured in the presence of 30 ng/ml of macrophage colony-stimulating factor (M-CSF) (PeproTech; Rocky Hill, NJ, USA) and 50 ng/mL of receptor activator of nuclear factor kappa-B ligand (RANKL) (PeproTech) for 4 days [26]. The animal research procedures were approved by the Animal Care and Use Committee of the Ajou University School of Medicine (IACUC No. 2014-0066), and all experiments were conducted in accordance with the institutional guidelines established by the Committee. All efforts were made to minimize animal suffering, and to reduce the number of mice used.

### Western blot analysis

The MC3T3-E1 cells and primary monocytes were lysed in RIPA buffer (150 mM NaCl, 1% Nonidet P-40, 0.5% sodium deoxycholate, 0.1% SDS, and 50 mM of Tris buffer, pH 8.0). The lysates were centrifuged at 16,000×g for 20 min at 4° C to remove cellular debris. The protein concentration was determined using the Dc Protein assay kit (BIO-RAD; Hercules, CA, USA). Protein samples were heated at 95° C for 5 min and analyzed by SDS-PAGE on 12% polyacrylamide gels. The proteins were electroblotted onto PVDF membrane (Millipore; Concord Road Billerica, MA, USA). The membrane blots were blocked with 5% (w/v) BSA, incubated with primary and secondary antibodies, and then visualized by the WEST-ZOL plus ECL Western blotting detection system (iNtRON Biotechnology; Daejeon, Korea). Anti-SQRDL antibody was purchased from Abcam (Cambridge, UK) and anti-β-actin, HRP-conjugated goat anti-rabbit IgG, and HRP-conjugated rabbit anti-goat IgG antibodies were purchased from Santa Cruz Biotechnology (Santa Cruz, CA, USA).

### Alkaline phosphatase (ALP) assay, ALP staining, and tartrate-resistant acid phosphatase (TRAP) assay

ALP activity was measured using a TRACP & ALP assay kit (Takara; Shiga, Japan). Total cell lysates were incubated at 37° C for 30 min in a buffer containing 1 mmol/L of Tris–HCl (pH 8.8), 0.5% Triton X-100, 10 mmol/L of Mg^2+^, and 5 mmol/L of p-nitrophenylphosphate as substrates, and the reaction was stopped by 0.5 N NaOH. The absorbance was read at 405 nm with a microplate reader (BIO-RAD). For ALP staining, cultured cells were fixed by drying for 10 min, washed twice with saline, and incubated with BCIP/NBT (Sigma-Aldrich) for 30 min in dark.

The osteoclast differentiation of the monocytes was assessed based on TRAP activity using TRACP and ALP assay kits (Takara). TRAP staining was assessed with an acid-phosphatase kit (Sigma-Aldrich).

### Assessment of *in vitro* bone mineralization

Mineralization of the MC3T3-E1 cells was assessed on day 14. The colonies were fixed with 70% ethanol for 10 min at room temperature, rinsed with water, and then stained with 40 mM of Alizarin red S (ARS) (Sigma-Aldrich). For quantification of the mineralized nodules, the stained ARS was extracted with 10% cethylpyridinium chloride, and its absorbance was read at 562 nm with a microplate reader (BIO-RAD).

### Quantitative reverse-transcription PCR (qRT-PCR)

Total RNA was extracted from the MC3T3-E1 cells using a TRIzol reagent (Invitrogen) according to the manufacturer’s instructions and quantified with a spectrophotometer (Beckman Coulter; Brea, CA, USA). The extracted RNA was subsequently reverse transcribed using a First-Strand cDNA Synthesis Kit (Fermentas; Hanover, MD, USA) with oligo (dT)_15–18_ and random hexamer primers. qRT-PCR was performed using the Quiagen Rotor-Gene Q (Quiagen; Hilden, Germany) and the ABI Prism 7500 Sequence Detection System (Life Technology). PCR amplifications (40 cycles) were performed in a total volume of 10 μl containing 100 ng OF cDNA using the Rotor-Gene SYBR Green PCR Kit (Quiagen) according to the manufacturer’s recommendation.

The specific primers used were as follows: 5′-TAA AGT GAC AGT GGA CGG TCC C-3′ and 5′-TGC GCC CTA AAT CAC TGA GG-3′ for the mouse *Runx2* (Genbank: NM_009820.5), 5′-ATG GCG TCC TCT CTG CTT G-3′ and 5′-TGA AAG GTC AGC GTA TGG CTT-3′ for the mouse *Sp7* (GenBank: NM_130458.3), 5′-TAG TGA ACA GAC TCC GGC GCT-3′ and 5′-TGT AGG CGG TCT TCA AGC CAT-3′ for the mouse *Bglap* (Genbank: NM_007541), and 5′-TGA CCA CAG TCC ATG CCA TC-3′ and 5′-GAC GGA CAC ATT GGG GGT AG-3′ for the mouse *Gapdh* (GenBank: NM_001289726.1). By normalizing to mouse *Gapdh*, relative quantification of gene expression was performed using the comparative threshold (Ct) method previously described [27].

### Statistical analysis

The statistical analysis in the association study was performed using PLINK version 1.07 (http://pngu.mgh.harvard.edu/~purcell/plink) and PASW statistics software, version 17.0 (SPSS Inc., Chicago, IL, USA). Significant differences in the characteristics between the controls and cases in Table 1 were determined by the two-tailed Student’s *t*-test. A logistic regression analysis was performed for the osteoporosis case-control association analysis. To minimize the effects of age and BMI differences between the case and control groups, all logistic regression analyses were adjusted for age and BMI as covariates. Residential area (Ansan and Ansung cities) was also included as a covariate. The estimated sample sizes for 80% study power at α = 0.05 were based on the KARE parameters, including minor allele frequency, effect size, odds ratio and the osteoporosis prevalence of Korean women using the Quanto program (version 1.2) (http://biostats.usc.edu/Quanto.html). The SNP Annotation and Proxy Search (SNAP) database (http://www.broadinstitute.org/mpg/snap/) was used for regional association plot drawing.

In the experimental studies, all the experiments were repeated independently at least three times, unless stated otherwise, and the results were presented as the means ± standard deviation (SD), as indicated. Statistical analyses were performed with PASW Statistics, version 17.0 (SPSS Inc.). Statistical significance between the groups was calculated with a Student’s t-test. A probability value (*p*) less than 0.05 (p<0.05) was considered statistically significant.

## Results

### GWA analysis of nsSNPs for osteoporosis in postmenopausal women

We performed an association analysis of 1180 nsSNPs for osteoporosis in 1,127 Korean postmenopausal women subjects in the KARE cohort. The logistic regression statistical analysis of the cases and controls was conducted based on the additive genetic model. We found 72 nsSNPs that showed a significant association with osteoporosis (*p*<0.05). No significant association signals satisfying the Bonferroni-corrected significance level, which was adjusted for multiple tests (*p*<4.23×10^-5^ calculated by 0.05/1180 SNPs), were observed in any of the tested nsSNPs. The top 10 nsSNPs showing the lowest *p*-values (*p* = 5.2×10^-4^–8.5×10^-3^) are summarized in Table 2. These nsSNPs were located in 10 distinct individual genes. No associations between these 10 genes and any osteoporosis-related phenotypes have been previously reported.

**Table 2:** List of top 10 nonsynonymous SNPs associated with osteoporosis in Korean postmenopausal women by logistic regression analysis.

nsSNP	Gene	Chr.	Base pair	A1	A2	MAF		Genotyping rate (%)	OR	CI 0.95	Add *p*	Sample size for 80% power	
						Case (n = 405)	Control (n = 722)					Case	Control
rs3013105	*LRRC38*	1	13674912	A	G	0.426	0.361	97.92	1.42	1.17–1.73	5.2 × 10^-4^	206	366
rs7970885	*OR10P1*	12	54317540	A	G	0.258	0.319	99.97	0.72	0.59–0.89	2.0 × 10^-3^	307	546
rs1044032	*SQRDL*	15	43755727	C	T	0.421	0.490	99.89	0.74	0.61–0.90	2.1 × 10^-3^	282	502
rs16995685	*DEFB127*	20	87576	T	G	0.390	0.457	99.29	0.75	0.62–0.92	4.4 × 10^-3^	317	564
rs3829767	*SYNE2*	14	63589208	T	C	0.159	0.204	100	0.70	0.55–0.90	4.6 × 10^-3^	377	671
rs4842838	*ADAMTSL3*	15	82373128	C	A	0.252	0.206	99.97	1.39	1.10–1.74	5.2 × 10^-3^	298	530
rs6746030	*SCN9A*	2	166807404	T	C	0.072	0.042	99.98	1.81	1.19–2.74	5.3 × 10^-3^	255	453
rs41481648	*NLRP8*	19	61159187	T	C	0.057	0.091	100	0.59	0.40–0.87	7.4 × 10^-3^	448	797
rs6759892	*UGT1A6*	2	234266408	G	T	0.216	0.260	100	0.73	0.58–0.92	8.1 × 10^-3^	378	673
rs2250860	*WFDC8*	20	43617789	G	A	0.017	0.034	100	0.42	0.22–0.80	8.5 × 10^-3^	583	1038

Age, BMI, and residential area were included as covariates in the additive genetic models. Sample size for 80% power at α = 0.05 is based on KARE parameters, including minor allele frequency of cases, odds ratio and the osteoporosis prevalence of Korean women. The SNP positions are based on the NCBI Build 36 human genome assembly. Abbreviations: A1, minor allele; A2, major allele; Add *p*, *p*-value in the additive genetic model; BP, base pair; Chr., chromosome; CI, confidence interval; MAF, minor allele frequency; OR, odds ratio; and SNP, single nucleotide polymorphism.

### *In silico* evaluation of the effects of the top 10 nsSNPs on protein function

**Fig 1:**
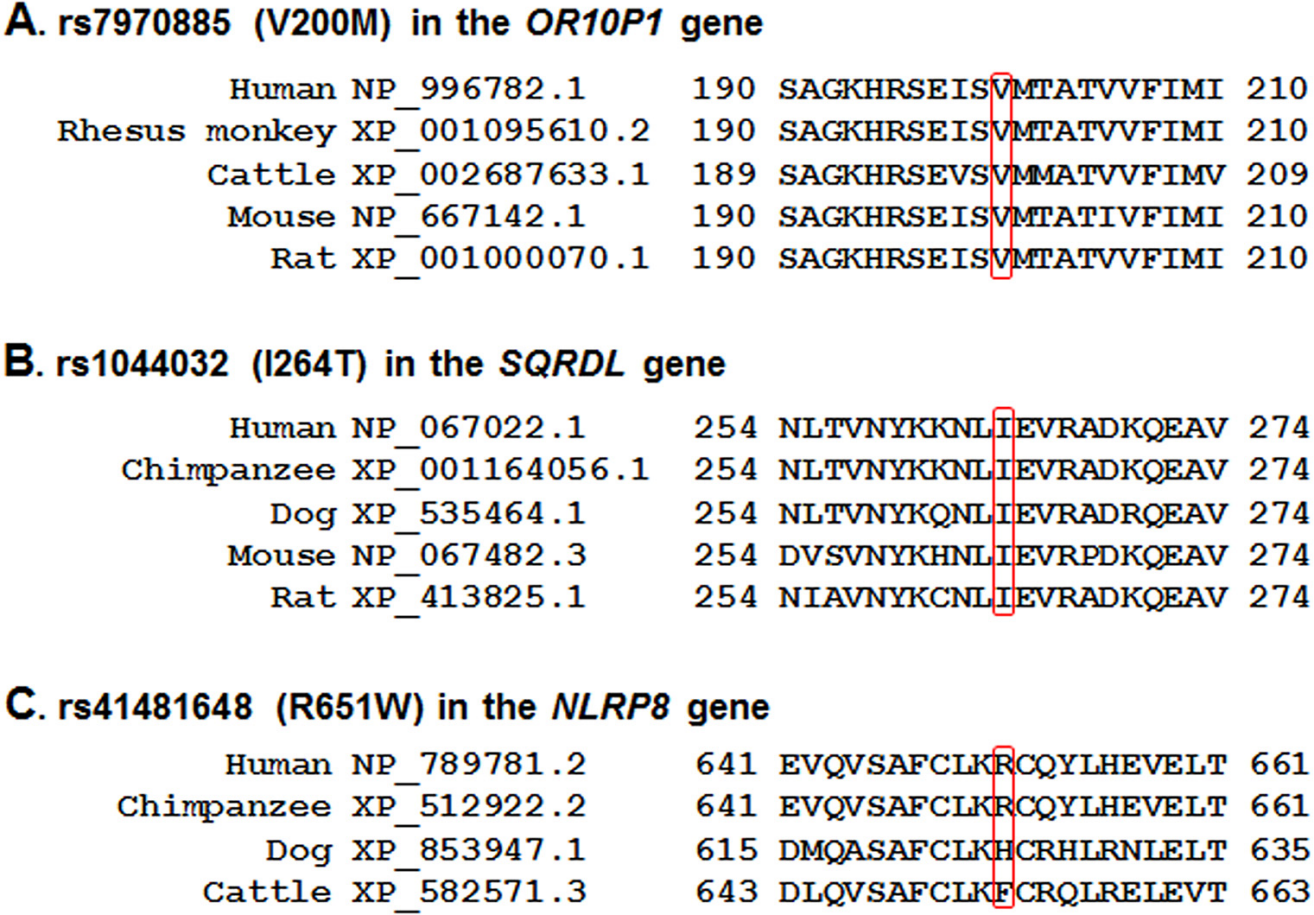
Comparison of the amino acid sequences in the polymorphism sites of the four genes among the species tested. Multiple sequence alignments of the amino acid sequences of the *OR10P1* (A), *SQRDL* gene (B), and *NLRP8* (C) are shown.

Next, to examine the functional effect of the amino acid alterations in the 10 nsSNPs on protein function, we conducted *in silico* prediction analysis using SIFT and PolyPhen-2 predicting methods. As shown in Table 3, three nsSNPs were predicted to have a deleterious effect on the protein function by at least one *in silico* predicting program: rs7970885 (V200M) in the *OR10P1* gene, rs1044032 (I264T) in the *SQRDL* gene, and rs41481648 (R651W) in the *NLRP8* gene. Comparison of the amino acid residues at the polymorphism sites of the three nsSNPs in the *OR10P1*, *SQRDL*, and *NLRP8* genes by multiple-sequence alignments of the orthologues revealed that the amino acid sequences of two of the nsSNPs, *OR10P1* rs7970885 (V200M) and *SQRDL* rs1044032 (I264T), were well conserved among all species compared (Fig 1).

**Table 3:** Summary of the nonsynonymous SNPs showing a deleterious effect on the protein’s function by *in silico* prediction analysis.

nsSNP	rs7970885	rs1044032	rs41481648
**Gene**	*OR10P1*	*SQRDL*	*NLRP8*
Gene ID	121130	58472	609659
Gene description	olfactory receptor, family 10, subfamily P, member 1	sulfide quinone reductase-like (yeast)	NLR family, pyrin domain containing 8
**mRNA**			
Genbank No.	NM_206899.1	NM_021199.3	NM_176811.2
cDNA position	598	791	1951
Nucleotide change	**G**TG > **A**TG	A**T**T > A**C**T	**C**GG > **T**GG
**Protein**			
Genbank No.	NP_996782.1	NP_067022.1	NP_789781.2
Position	200	264	651
Amino acid change	V [Val] > M [Met]	I [Ile] > T [Thr]	R [Arg] > W [Trp]
**Minor allele frequency**			
KARE controls	0.319	0.490	0.091
European	0.385	0.195	0.063
Han-Chinese	0.314	0.465	0.047
Japanese	0.192	0.453	0.047
Sub-Saharan African	0.093	0.124	0.142
**Protein damage prediction**			
SIFT	Tolerated	Damaging	Damaging
PloyPhen-2	Possibly Damaging	Benign	Probably Damaging

Minor allele frequencies were obtained from the NCBI dbSNP database (http://www.ncbi.nlm.nih.gov). Abbreviations: KARE, Korea Association Resource cohort; PolyPhen-2, Polymorphism Phenotyping v2; and SIFT, Sorting Intolerant From Tolerant

**Fig 2:**
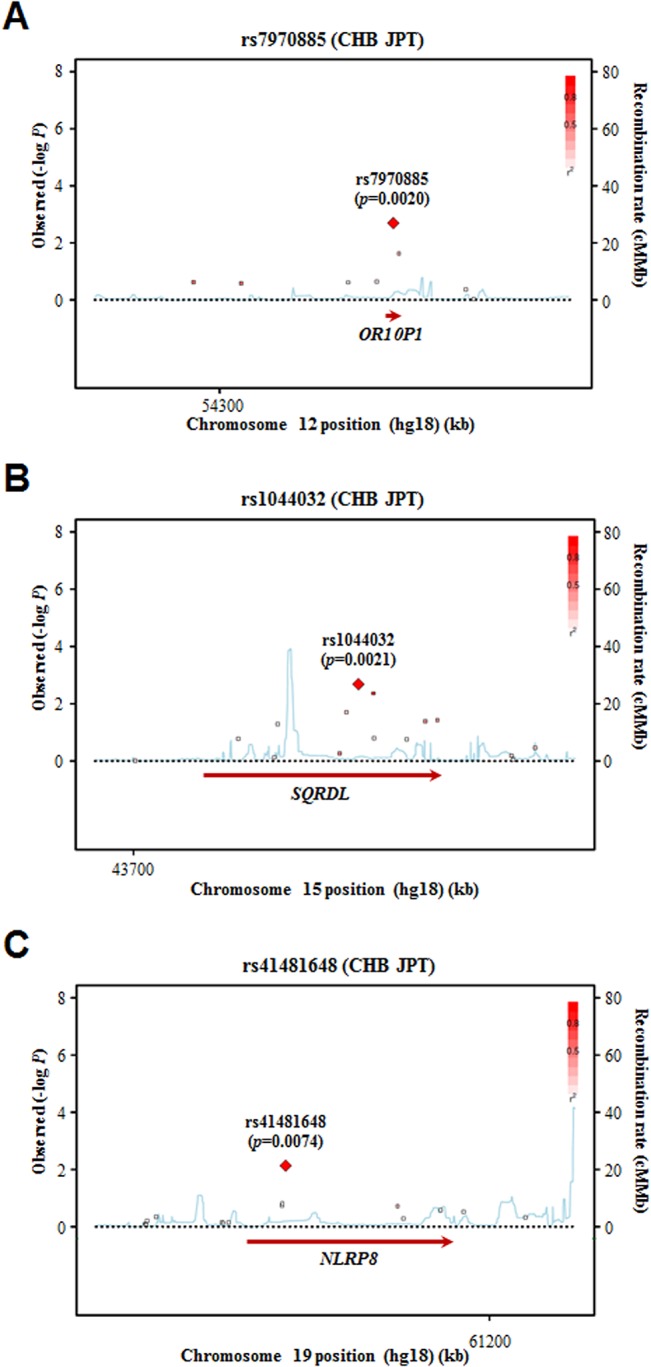
Plots of the association results of SNPs in the four osteoporosis-associated genes in the Korean postmenopausal women of the KARE cohort. The regional association plots of the *OR10P1* (A), *SQRDL* gene (B), and *NLRP8* (C) were generated using the SNAP database (http://www.broadinstitute.org/mpg/snap/). The statistical significance (–log_10_ *p*-value) of the analyzed SNPs is plotted. The red diamond with a SNP number and *p*-value represents the SNP most strongly associated with osteoporosis, and its correlated SNPs are shown in the indicated colors in accordance with the levels of linkage disequilibrium (LD) (*r* ^2^). The recombination rate estimated from the HapMap CHB and JPT population data is shown by a blue line. The position (Mb) of each gene on human chromosomes (NCBI build 36) is shown at the bottom.

Association signals can be highly reliable in cases multiple SNPs in the same gene are associated with the same diseases or traits. Hence, we investigated whether other significant SNPs were present in the vicinity of the four nsSNPs. Plots for the association results of all the SNPs in the three genes with osteoporosis are shown in Fig 2. Multiple SNPs in the *SQRDL* gene showed significant associations with osteoporosis (*p*<0.05). Two SNPs located in the intron region and two SNPs located downstream of the *SQRDL* gene were significantly associated with osteoporosis (*p* = 0.0044–0.041) (S1 Table).

Taken together, the rs1044032 nsSNP (c.790T>C, p.I264T) in the *SQRDL* gene, a hydrogen sulfide (H_2_S)-catalyzing sulfide quinone reductase-like protein, was selected as a primary candidate for further functional study. The results of the case-control association analysis of the rs1044032 nsSNP with osteoporosis revealed a *p*-value of 0.0021 and an odds ratio (OR) of 0.74 in the additive genetic model (Table 2).

### Effect of amino acid alterations corresponding to the *SQRDL* rs1044032 nsSNP on the differentiation of osteoblasts and osteoclasts

Next, we attempted to replicate experimentally the statistical association results of the *SQRDL* rs1044032 nsSNP. We investigated whether the alteration of the amino acid residue at the 264 position of the SQRDL protein (I264T) influenced osteoblast and/or osteoclast differentiation, which is essential for bone remodeling [1, 2, 4]. Two human SQRDL cDNA lentiviral constructs were generated: SQRDL_WT (wild type, isoleucine at position 264) and SQRDL_I264T (threonine at position 264). An empty lentiviral vector was used as a negative control.

**Fig 3:**
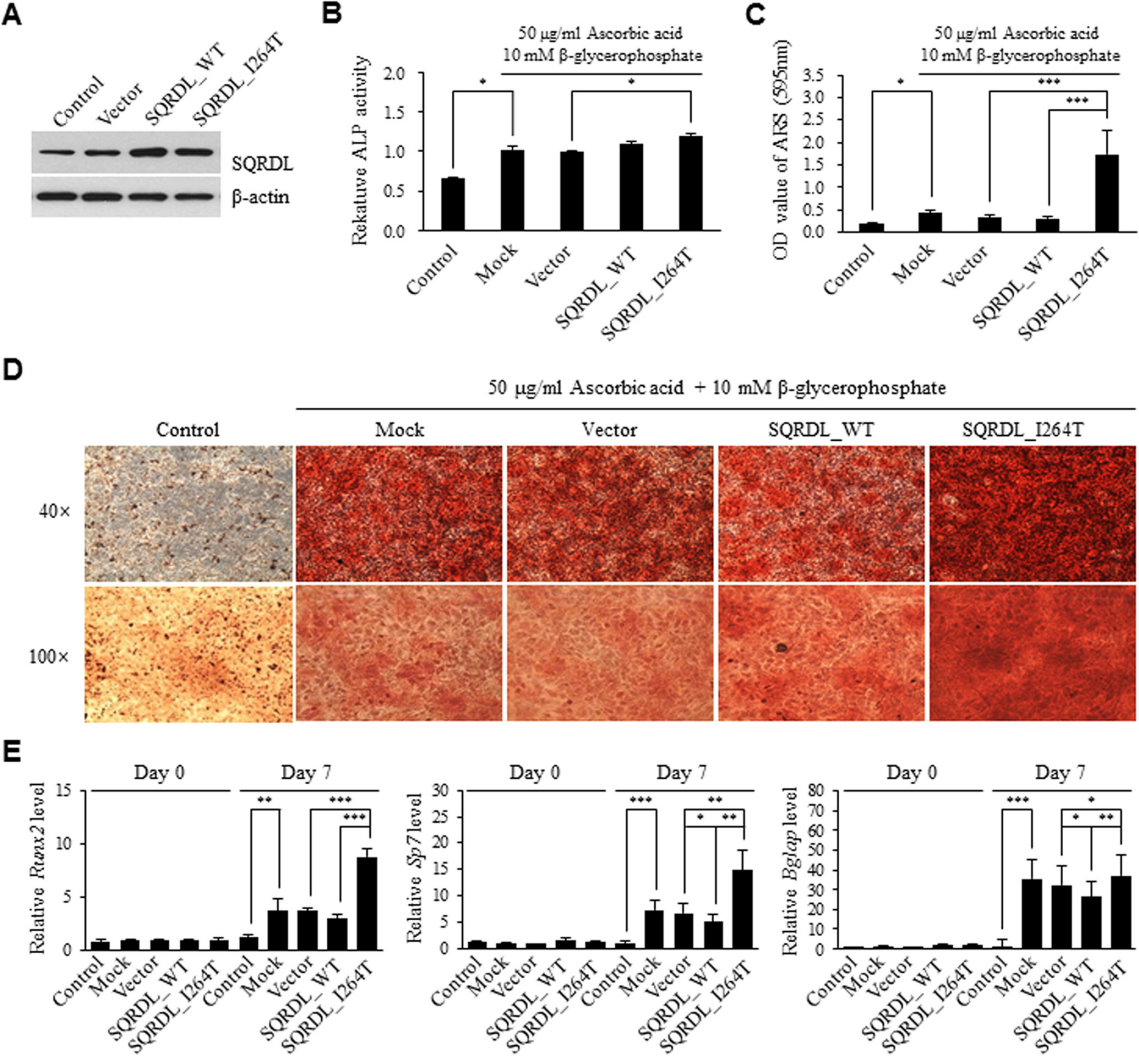
Effect of the SQRDL I264T variant on osteoblast differentiation. Preosteoblast MC3T3-E1 cells were infected with SQRDL_WT or SQRDL_I264T lentiviral constructs and then selected by puromycin for one week. Empty lentiviral vector-transfected and nontransfected MC3T3-E1 cells were used as controls. For induction of osteoblast differentiation, cells not overexpressing of any vectors or constructs (Mock), cells overexpressing the vector (Vector), or cells overexpressing two SQRDL constructs were treated with 50 μg/mL of ascorbic acid and 10 mM of β-glycerophosphate for the indicated period. (A) Validation of the overexpression of the SQRDL constructs in the lentiviral-infected MC3T3-E1 cells. The protein levels of SQRDL and β-actin were analyzed by Western blotting with the specific antibodies. (B) Assessment of the osteoblast differentiation level of the cells based on ALP activity. The cells were treated with osteoblast differentiation induction reagents for 3 days. The ALP activity was measured with an ALP assay kit, and the absorbance was read at 405 nm with a microplate reader. The results were expressed as the fold change over the control (vector). (C, D) Assessment of *in vitro* bone mineralization level of the cells. The cells were treated with osteoblast differentiation induction reagents for 14 days. They were stained with ARS, and the positively stained nodules were visualized under a microscope at a magnification of 40 and 100. The quantification of ARS staining was conducted by measuring the amount of ARS extracted using 10% cethylpyridinium chloride. (E) Quantification of the mRNA expression level of osteoblastogenesis markers. The cells were treated with osteoblast differentiation induction reagents for 7 days. Total RNA was isolated from the cells before treatment with induction reagents (Day 0) and 7 days after the treatment (Day 7). Quantitative RT-PCR was performed with gene-specific primers for *Runx2*, *Sp7*, *Bglap*, and *Gapdh* (relative control). The results were expressed as the fold change over the control (vector, Day 0). *p<0.05, **p<0.01, ***p<0.001.

We investigated the effect of the amino acid alteration on osteoblast differentiation. Mouse preosteoblast MC3T3-E1 cells were infected with two viral constructs, and antibiotics selection was done by treatment with 4 μg/mL of puromycin for 1 week. Similar levels of overexpression by the two constructs were confirmed by Western blotting (Fig 3A). The SQRDL protein was also detected in the noninfected (control) and in the empty lentiviral vector-infected (vector) MC3T3-E1 cells, indicating that the MC3T3-E1 cells endogenously expressed the *Sqrdl* gene (Fig 3A). The osteoblastic differentiation levels of the MC3T3-E1 cells expressing each construct were measured with an ALP activity assay after osteoblast induction by treatment with 50 μg/mL of ascorbic acid and 10 mM of β-glycerophosphate. Based on the result from the vector, the overexpression of SQRDL_WT did not affect ALP activity, whereas the overexpression of SQRDL_I264T significantly increased the ALP activity of the cells (Fig 3B). However, no significant differences between the two constructs were observed. For *in vitro* bone mineralization analysis, the MC3T3-E1 cells expressing each construct were treated with osteoblast induction reagents and cultured for 21 days. Mineralized nodule formation of the cells was detected by microscopic observation of the nodules after ARS staining, and the level of ARS was quantified by measuring the amount rinsed from the stained nodules. Overexpression of SQRDL_WT did not affect the mineralization of the cells, whereas overexpression of SQRDL_I264T significantly increased the mineralization of the cells (Fig 3C and 3D). In addition, the degree of mineralization was significantly higher in the cells overexpressing SQRDL_I264T than in those with SQRDL_WT (Fig 3C and 3D). We further examined the mRNA expression levels of three genes, *Runx2* (Runt-related transcription factor 2), *Sp7* (Osterix), and *Bglap* (Osteocalcine), that encode the key regulators of osteoblast differentiation [28–30]. The MC3T3-E1 cells expressing each construct were treated with osteoblast differentiation induction reagents for 7 days, and the mRNA expression of the three genes was examined by qRT-PCR with gene-specific primers. The mRNA expression levels were significantly higher in the cells overexpressing SQRDL_I264T than in those with SQRDL_WT (Fig 3E). These results demonstrated that SQRDL I264T variation was closely involved in the osteoblast differentiation of the MC3T3-E1 cells.

Next, we investigated the effect of the amino acid alteration on osteoblast differentiation. Mouse primary-cultured monocyte cells derived from mouse bone marrow were infected with two viral constructs. For induction of osteoblast differentiation, monocyte cells expressing each constructs were cultured for 5 days in the presence of 30 ng/mL of M-CSF and 50 ng/mL of RANKL. The results of a TRAP activity assay and TRAP staining revealed no significant differences among the vector, SQRDL_WT, and SQRDL_I264T (S1 Fig), suggesting that the SQRDL I264T variation did not affect osteoblast differentiation of the monocytes.

## Discussion

A large number of susceptibility genes/loci for osteoporosis and/or osteoporosis-related traits have been identified, mainly by candidate gene-based association studies and GWA studies [7, 13, 14, 31–33]. However, only a small portion of these have experimentally investigated the functional mechanisms of these genes/loci in bone remodeling [7]. As the effect size of most common variants in diseases is small or modest [34], it is difficult to prove the role of genetic variants in disease phenotypes. It is more difficult to determine their functional effects if they are located on noncoding intron regions of genes or intergenic regions. In this respect, nsSNPs conferring amino acid changes are advantageous because they permit experimental validation of the statistically identified genetic variants. So far, a few nsSNPs have been reported to be associated with osteoporosis-related phenotypes: three variants (A1330V, Q89R and V667M) of the *LRP5* gene were associated with BMD in many ethnic cohorts [12], an A152V variant of the *BMP4* gene was associated with bone mass in Caucasian women [35], and an N19L variant of the *CLEC2D* gene was associated with BMD in Spanish women [36]. In addition, three variants (R47S, P108L, and I194V) of the *WDSOF1* gene were associated with total body BMD in Japanese postmenopausal women [37], R325Q in the *GGCX* gene was associated with BMD in elderly Japanese women [38], an A65G variant of the *CER1* gene was associated with BMD and fracture in Chinese women [39], and an Q223R variant of the *LEFR* gene was associated with BMD in Danish postmenopausal women [40]. Finally, L109R and Q223R variants of the *LEFR* gene were associated with BMD in Korean women [41], and two nsSNPs (S136C and S704A) in the *EIF2AK3* gene were associated with BMD in Amish subjects [42].

Among the aforementioned variants, only the *LRP5* and *LEFR* nsSNPs were associated with BMD in multiple populations. The other nsSNPs were identified in a single ethnic cohort. With regard to genetic variants for osteoporosis-related phenotypes, replicated association studies of the discovered nsSNPs in other ethnic cohorts have rarely been reported. In this study, to discover novel nsSNP(s) associated with osteoporosis in Korean postmenopausal women in the KARE cohort, we conducted a GWA analysis of all genotyped whole nsSNPs for osteoporosis in a case-control association study. In the same KARE cohort, a GWA study of whole SNPs for bone density at the radius, tibia, and heel was previously carried out [19]. That study identified two noncoding SNPs in the *FAM3C* and *SFRP4* genes that were significantly associated with BMD [19]. However, there have been no reports of nsSNPs associated with osteoporosis in the KARE cohort. Therefore, this is the first study focused on the association analysis between nsSNPs and osteoporosis in the KARE cohort.

In our association analysis of 1180 nsSNPs, 72 nsSNPs displayed a significant association with osteoporosis (*p*<0.05). No nsSNPs satisfied the Bonferroni-corrected significance level, adjusted for multiple tests. Because the previously identified nsSNPs having moderately significant *p*-values showed an obvious impact on osteoporosis-related phenotypes [12, 35–42], some of our discovered nsSNPs were anticipated to be meaningful genetic variants involved in osteoporosis etiology. We focused on the 10 best nsSNPs based on the *p*-value levels (Table 2). As the 10 genes corresponding to the 10 nsSNPs were not previously speculated to be associated with osteoporosis and/or osteoporosis-related phenotypes, we first investigated whether the association signals of these nsSNPs were real. We could not perform a replication analysis of our association results due to the lack of available cohorts. Therefore, we performed an *in silico* prediction analysis of the 10 nsSNPs with SIFT and PolyPhen-2 predicting methods. Both methods are commonly used for *in silico* prediction of protein function based on single amino acid changes [18, 25]. Three nsSNPs were predicted to have a deleterious effect on the corresponding protein function (Table 3). Following a sequence conservation evaluation of the amino acid residues at the position of these three nsSNPs among orthologues in other species, we selected two nsSNPs in the *OR10P1* and *SQRDL* genes as meaningful candidates (Fig 1). Besides nsSNPs, an association analysis of SNPs located in the regulatory regions or noncoding intron regions of the four genes revealed that multiple SNPs in the *SQRDL* gene were associated with osteoporosis in KARE postmenopausal women (Fig 2). Finally, we selected one nsSNP in the *SQRDL* gene, SQRDL I264T (rs1044032), as a genetic variant for a further functional study.

Overexpression of the SQRDL WT and SQRDL I264T variant in the preosteoblast MC3T3-E1 cells revealed that the SQRDL I264T variant had a significantly positive influence on bone remodeling, particularly osteoblast differentiation, whereas the SQRDL WT had no effect or a negative effect on osteoblast differentiation. The aforementioned was demonstrated using orthodox methods for evaluating osteoblast differentiation, including ALP activity, mineralization, and mRNA expression of osteoblast differentiation markers, *Runx2*, *Sp7*, and *Bglap* genes (Fig 3) [28–30, 43]. Importantly, these results are consistent with those of the association analysis. In the case-control association analysis of rs1044032 nsSNP and osteoporosis, the OR was 0.74 in the additive genetic model (Table 2). An OR lower than 1.0 indicates that subjects who have the effect allele (minor allele ‘C,’ SQRDL I264T variant) in the rs1044032 *SQRDL* nsSNP might exhibit less susceptibility to osteoporosis than those with the non-effect allele (major allele ‘T,’ SQRDL I264 WT) in the nsSNP. Likewise, the osteoblast differentiation of the preosteoblst MC3T3-E1 cells overexpressing the SQRDL I264T variant was significantly better than that of the cells overexpressing the WT SQRDL (Fig 3). These results strongly suggest that the SQRDL I264T nsSNP may be a significant susceptibility variant for osteoporosis in Korean postmenopausal women, with a large effect size, and that it may be involved in osteoblast differentiation.

SQRDL, a sulfide quinone reductase-like protein or a sulfide:quinone oxidoreductase, is a vertebrate homolog of bacterial sulfide-quinone oxidoreductase and fission yeast heavy metal tolerance 2 protein [44–48]. SQRDL is expressed in mitochondria and has a role in regulating cellular hydrogen sulfide (H_2_S) levels by catalyzing the oxidation of H_2_S at the first step of the mitochondrial H_2_S metabolism process.[46–48] The SQRDL protein is expressed in multiple tissues [46, 47]. In the human protein atlas web site (http://www.proteinatlas.org), the SQRDL mRNA and protein are expressed in bone marrow tissue. Our results also show that mouse preosteoblast MC3T3-E1 cells express quite a large amount of SQRDL protein (Fig 3A). These data point to an important role for SQRDL in osteoblast lineage cells. The results of our association and experimental studies support this hypothesis. H_2_S is considered a toxic compound. However, a recent study demonstrated its role as an important gasotransmitter in multiple inter- and intracellular signaling pathways and as a metabolic, inflammatory, neuro, and vascular modulator [49]. In bone remodeling, H_2_S inhibited osteoblastic transformation and mineralization of human vascular smooth muscle cells (VSMCs) [50]. Treatment of VSMCs with H_2_S significantly decreased ALP activity, osteocalcin protein expression, and mineralization in a dose-dependent manner, pointing to a toxic effect of H_2_S on the osteoblast differentiation of VSMCs [50]. On the contrary, another study reported that H_2_S inhibited dexamethasone-induced osteoblast damage [51]. Two H_2_S-producing enzymes, cystathionine β-synthase and cystathionine γ-lyase (CSE), were significantly downregulated in dexamethasone-induced damaged MC3T3-E1 cells [51]. In addition, H_2_S was reported to inhibit osteoclast differentiation [52], whereas CSE was reported to accelerate osteoclast differentiation [53]. Collectively, the cellular H_2_S level is a critical factor in regulation of bone remodeling. However, further studies are required to clarify the beneficial and harmful effect of H_2_S on bone remodeling and on other cellular signals [54]. The known effects of H_2_S on bone remodeling may explain the findings of the current study, which demonstrated a significant effect of the H_2_S-catalyzing enzyme SQRDL I264T variant on osteoblast differentiation of the preosteoblst MC3T3-E1 cells.

A previous GWA study of 1792 Filipino women reported that the rs12594514 SNP in the *SQRDL* gene was associated with two obesity-related phenotypes: weight (β = 1.705, *p* = 5.28×10^-6^) and waist (β = 1.727, *p* = 7.16×10^-6^) [55]. As our genotyping data in the KARE cohort did not include this SNP, we performed an imputation analysis of this rs12594514 SNP, using SNAP version 2.2 (SNP Annotation and Proxy Search, http://www.broadinstitute.org/mpg/snap/). The rs11070467 SNP located in the 3ʹ downstream region of the *SQRDL* gene was located closest to the rs12594514 SNP and showed the strongest correlation with this SNP (distance: 70 bp and *R* ^2^ = 0.983). This rs11079467 SNP showed a significant association with osteoporosis in our postmenopausal cohort (*p*-value = 0.041, OR = 1.22, and CI = 1.01–1.49) (S1 Table). Emerging evidence demonstrating the correlation between obesity and osteoporosis [56], together with the fact that progenitor cells of adipocytes and osteoblasts originate from common mesenchymal stem cells [57], may support our findings describing a new role of SQRDL in osteoblast differentiation.

In conclusion, in a GWA study of 1180 nsSNPs, we discovered a novel SQRDL I264T nsSNP, which served as a significant susceptibility variant in osteoporosis among Korean postmenopausal women. Furthermore, we experimentally demonstrated that this genetic variant was closely involved with the underlying mechanism of bone formation and that it had a positive effect on osteoblast differentiation of preosteoblast cells.

## Supporting Information

S1 Fig Effect of the SQRDL I264T variant on osteoclast differentiation. Primary monocytes were infected with SQRDL_WT or SQRDL_I264T lentiviral constructs. Empty lentiviral vector-transfected and nontransfected monocyte cells were used as controls. (A) Validation of successful isolation of monocytes from mouse bone marrow. Primary-cultured monocytes were identified by immunophenotypic analysis with a monocyte-specific surface positive marker (PE-conjugated CD11b antibody). The absence of contamination of MSCs was confirmed by an immunophenotypic analysis with an MSC-positive marker (PE-conjugated CD90 antibody) using FACS analysis. (B) Validation of the overexpression of the SQRDL constructs in the lentiviral-infected monocyte cells. Three days after lentiviral infection, the protein levels of SQRDL and β-actin were analyzed by Western blotting with specific antibodies (C, D) Assessment of the osteoclast differentiation level of the monocyte cells. For induction of osteoblast differentiation of monocytes, monocyte cells not overexpressing any vectors (Mock), cells overexpressing the vector (Vector), or two SQRDL constructs were cultured in the presence of 30 ng/mL of M-CSF and 50 ng/mL of RANKL for 5 days. A TRAP activity of the cells was performed, and the results were expressed as the fold change over the control (vector). The cells also stained with a TRAP staining kit, and the differentiated osteoclast cells were visualized under a microscope at a magnification of 40 and 100. *p<0.05.
(TIF)

S1 Table Result of the case-control association analysis of SNPs in the *SQRDL* gene with osteoporosis in the Korean postmenopausal women subjects. Age, BMI, and residential area were included as covariates in the additive genetic models. *P*-values < 0.05 are indicated in bold and the nonsynonymous SNP is indicated in red. The SNP positions are based on the NCBI Build 36 human genome assembly. Abbreviations: A1, minor allele; Add *p*, *p*-value in the additive genetic model; CI, confidence interval; MAF, minor allele frequency; OR, odds ratio; and SNP, single nucleotide polymorphism.
(XLSX)
