# Supplementary figures and images for: Association of the I264T Variant in the Sulfide Quinone Reductase-Like (SQRDL) Gene with Osteoporosis in Korean Postmenopausal Women
Source: PLoS One. 2015 Aug 10;10(8):e0135285. doi: 10.1371/journal.pone.0135285 (PMC4530967; doi:10.1371/journal.pone.0135285)

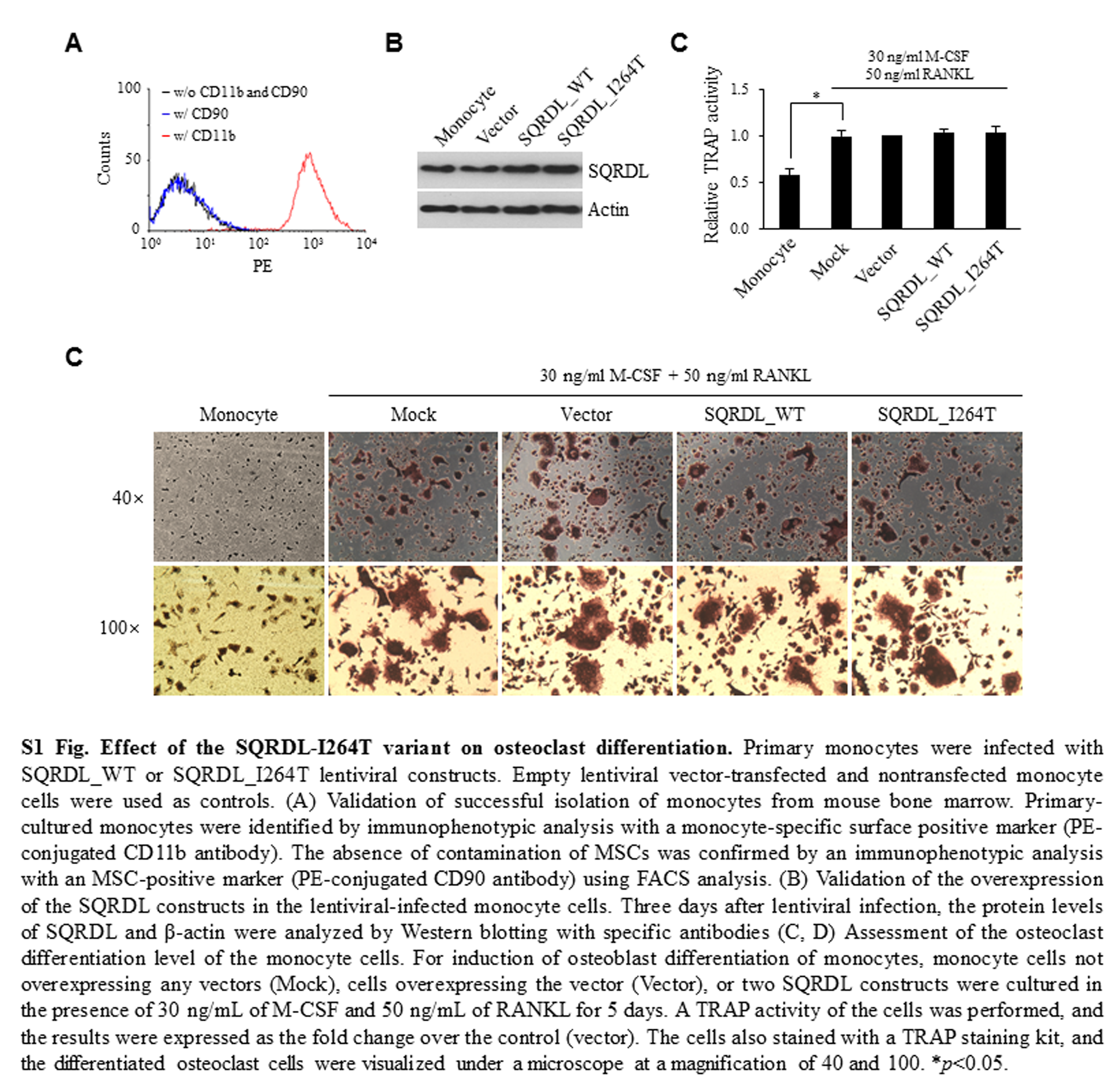

Supplement: S1 Fig — Primary monocytes were infected with SQRDL_WT or SQRDL_I264T lentiviral constructs. Empty lentiviral vector-transfected and nontransfected monocyte cells were used as controls. (A) Validation of successful isolation of monocytes from mouse bone marrow. Primary-cultured monocytes were identified by immunophenotypic analysis with a monocyte-specific surface positive marker (PE-conjugated CD11b antibody). The absence of contamination of MSCs was confirmed by an immunophenotypic analysis with an MSC-positive marker (PE-conjugated CD90 antibody) using FACS analysis. (B) Validation of the overexpression of the SQRDL constructs in the lentiviral-infected monocyte cells. Three days after lentiviral infection, the protein levels of SQRDL and β-actin were analyzed by Western blotting with specific antibodies (C, D) Assessment of the osteoclast differentiation level of the monocyte cells. For induction of osteoblast differentiation of monocytes, monocyte cells not overexpressing any vectors (Mock), cells overexpressing the vector (Vector), or two SQRDL constructs were cultured in the presence of 30 ng/mL of M-CSF and 50 ng/mL of RANKL for 5 days. A TRAP activity of the cells was performed, and the results were expressed as the fold change over the control (vector). The cells also stained with a TRAP staining kit, and the differentiated osteoclast cells were visualized under a microscope at a magnification of 40 and 100. *p<0.05. (TIF) [file pone.0135285.s001.tif]
